# The effect of PTZ-induced epileptic seizures on hippocampal expression of PSA-NCAM in offspring born to kindled rats

**DOI:** 10.1186/1423-0127-19-56

**Published:** 2012-05-31

**Authors:** Aliakbar Rajabzadeh, Alireza Ebrahimzadeh Bideskan, Alireza Fazel, Mojtaba Sankian, Houshang Rafatpanah, Hossein Haghir

**Affiliations:** 1 Department of Anatomy and Cell Biology, Mashhad, Iran; 2 Bu-ali Research Institute, Immunology Research Center, Mashhad, Iran; 3 Inflammation and inflammatory Diseases Research Center, School of Medicine, Mashhad University of Medical Sciences (MUMS), Mashhad, Iran; 4 Department of Anatomy and Cell Biology, School of Medicine, Mashhad University of Medical Sciences, Azadi Sq., Vakilabad Blvd, P.O.Box 91779-48564, Mashhad, Iran

**Keywords:** Maternal Seizure, Polysialylated Neural Cell Adhesion Molecule, Kindling, Rat Hippocampus

## Abstract

**Background:**

Maternal epileptic seizures during pregnancy can affect the hippocampal neurons in the offspring. The polysialylated neural cell adhesion molecule (PSA-NCAM), which is expressed in the developing central nervous system, may play important roles in neuronal migration, synaptogenesis, and axonal outgrowth. This study was designed to assess the effects of kindling either with or without maternal seizures on hippocampal PSA-NCAM expression in rat offspring.

**Methods:**

Forty timed-pregnant Wistar rats were divided into four groups: A) Kind^+^/Seiz^+^, pregnant kindled (induced two weeks prior to pregnancy) rats that received repeated intraperitoneal (i.p.) pentylenetetrazol, PTZ injections on gestational days (GD) 14-19; B) Kind^-^/Seiz^+^, pregnant non-kindled rats that received PTZ injections on GD14-GD19; C) Kind^+^/Seiz^-^, pregnant kindled rats that did not receive any PTZ injections; and D) Kind^-^/Seiz^-^, the sham controls. Following birth, the pups were sacrificed on PD1 and PD14, and PSA-NCAM expression and localization in neonates’ hippocampi were analyzed by Western blots and immunohistochemistry.

**Results:**

Our data show a significant down regulation of hippocampal PSA-NCAM expression in the offspring of Kind^+^/Seiz^+^ (*p* = 0.001) and Kind^-^/Seiz^+^ (*p* = 0.001) groups compared to the sham control group. The PSA-NCAM immunoreactivity was markedly decreased in all parts of the hippocampus, especially in the CA3 region, in Kind^+^/Seiz^+^ (*p* = 0.007) and Kind^-^/Seiz^+^ (*p* = 0.007) group’s newborns on both PD1 and 14.

**Conclusion:**

Our findings demonstrate that maternal seizures but not kindling influence the expression of PSA-NCAM in the offspring’s hippocampi, which may be considered as a factor for learning/memory and cognitive impairments reported in children born to epileptic mothers.

## Background

Epilepsy is one of the most common neurological disorders (0.5–2% of the general population). It is estimated that there are more than seven million pregnant women suffering from epilepsy in the world [1,2], and that about 1% of all pregnant women have experienced seizure during their pregnancy [3]. Although most babies born from epileptic mother are normal, they are exposed to a higher risk of central nervous system (CNS) abnormalities [4,5]. Generalized seizures could lead to irreparable damages in the brain and also other organs. The two major mechanisms contributed to these damages include hypoxia and acidosis [6,7]. Several morphological changes such as neural cell shrinkage, nuclear pyknosis, and karyorrhexis in the rat pups hippocampus born from epileptic mothers having seizures during pregnancy have been reported previously [8]. A recent study has shown that maternal seizures during pregnancy cause severe cognitive disturbances and decrease in learning and memory in the offspring [9]. In addition, it seems that some of the learning and memory disorders in adolescence and adulthood are probably due to neurogenesis impairment, neuronal migration failure, impaired hippocampal maturation, and neurodegeneration during the embryonic period [9-11].

The majority of the cortical neurogenesis in rodents occurs during the 2^nd^ and 3^th^ week of prenatal period; and in rats, formation of the hippocampus is completed during the first 2 weeks of postnatal life [12,13]. It is well known that different stages of brain development are controlled by various factors such as growth factors, signaling proteins, and cell adhesion molecules; and that changes in these factors might lead to neuro-developmental impairments [14,15]. One of the molecules especially found in the CNS is the polysialylated neural cell adhesion molecule, PSA-NCAM [16,17]. PSA-NCAM is highly expressed in developing and migrating neurons especially during prenatal and early-postnatal neurodevelopment [18,19]. This molecule plays a critical role in neural plasticity, axonal outgrowth, neurogenesis, synaptogenesis; and also in several phenomena such as recognition, learning, and spatial memory [16,20,21]. Furthermore, elimination of PSA causes impairment in neural cell migration, neurite sprouting, synaptogenesis, and learning/memory [22-24].

Kindling is an experimental model for epilepsy; and pentylenetetrazol (PTZ) is a GABA_A_ receptor antagonist commonly used as a convulsing drug in experimental studies [25]**.** The PTZ-induced kindling was first described by Mason and Cooper in rats (**1972**). This phenomenon is characterized by an increased susceptibility to seizures after repeated injections of PTZ [26].

Sato and colleagues have shown that PTZ-induced seizures increase the number of PSA-NCAM positive cells in the hippocampus of kindled rats [27]. It has been reported that kainate-induced seizures cause neuronal loss in hippocampus [28]. However, the effects of maternal seizures on PSA-NCAM expression in the hippocampus of the offspring are unknown.

Here, using western blotting and immunohistochemistry techniques, we have evaluated the effects of PTZ-induced epileptic seizures on PSA-NCAM expression and its distribution in developing rat hippocampus born to kindled and non-kindled mothers on PD1 and PD14.

## Methods

This experimental research was done during 2009 in Mashhad University of Medical Sciences according to ethics committee guide lines including The National Institutes of Health(NIH) and all protocols of animal experiments have been approved by the Institution's Animal Care Committee.

### Animals

Forty Wistar rats (8 week-old, weighing 180–200 g) were purchased from the animal center laboratory of Mashhad University of medical sciences, in Mashhad, Iran. The animals were maintained at the animal house under controlled conditions (12 hr light-and-dark cycles, at 21 °C with 50% relative humidity) with laboratory chow and water provided *ad libitum.* Before mating, the female rats were divided into two groups: Kindled and Non-Kindled (N = 20 each).

### Kindling procedure

For kindling, female rats received a single dose of 40 mg/kg PTZ (Sigma, USA) dissolved in 1 ml of normal saline intraperitoneally (i.p.), every 48 hr. A total of 12-15 doses of PTZ were given to each rat. The convulsive behavior was observed for 20 min after each PTZ-injection. The seizures were classified according to the Racine score [29] as follows: stage 0, no response; stage 1, ear and facial twitching; stage 2, myoclonic jerks without upright position; stage 3, myoclonic jerks, upright position with bilateral forelimb clonus; stage 4, tonic–clonic seizures; stage 5, generalized tonic–clonic seizures, loss of postural control. To check the maintenance of kindling state, the animals were challenged with a sub-convulsive PTZ dose (40 mg/kg) 10 days after the last kindling injection [30]. Only the rats showing generalized tonic–clonic seizures were used as kindled (Kind^+^). Rats that received no PTZ before pregnancy were used as non-kindled (Kind^-^), and rats injected with equal volumes of normal saline (the solvent of PTZ) were used as sham controls (Kind^-^/Seiz^-^).

### Breeding protocol and study groups

Two weeks after kindling confirmation, kindled and non-kindled female rats were placed with males in the late afternoon (4-5 PM) and removed the next morning (9-10 AM). The day on which spermatozoa were found in the vaginal smear was designated as embryonic day 0 (E0). After pregnancy confirmation, rats were randomly divided into four groups as follows (N = 10 in each group): Group A, pregnant kindled rats that received i.p. PTZ injections (40 mg/kg) during pregnancy from Gestational Days (GD) 14 to 19 every 48 hr (Kind^+^/Seiz^+^); Group B, pregnant non-kindled rats that received i.p. PTZ injections (40 mg/kg) during pregnancy from GD14 to GD19 every 48 hr, exhibiting generalized tonic–clonic seizures (Kind^-^/Seiz^+^); Group C, pregnant kindled rats that did not receive any PTZ injection during pregnancy (Kind^+^/Seiz^-^); and Group D, pregnant non-kindled rats injected by normal saline with equal volume of PTZ during pregnancy from GD14 to GD19 every 48 hr (the Sham control = Kind^-^/Seiz^-^).

### Western blotting

Following anesthesia and craniotomy, rat offspring’s brain (N = 5 for each group) were removed on PDs 1 and 14. The hippocampi were isolated carefully and stored at -80 °C. In the next step, 50 mg of the hippocampus tissue from each rat was homogenized in 1 ml of lysis buffer containing Tris–HCl (0.3027 gr), NaCl (0.4383 gr), EDTA (0.0186 gr), Triton 100× (0.5 ml), and a protease inhibitor cocktail (Roche, Germany). Homogenized tissues were centrifuged at 12,300 g for 20 minutes at 4 °C. The proteins of each sample were electrophoresed under non-reducing conditions for 1 hr at 140 constant voltages on a 10% SDS-polyacrylamide gel. The protein mixture was then transferred from the gel onto a PVDF membrane (Millipore, Bedford, MA) using a Bio-Rad trans-blot apparatus at 300 mA for 15 minutes. Blots were blocked with TBS containing 5% skim milk at 4 °C for 12 hr. After washing the blots with TBS-T, blots were incubated with anti-PSA-NCAM mouse IgM monoclonal antibody as the primary antibody (diluted 1:5000 in 5% TBS-Skim milk) at 4 °C overnight. Following three washes with TBS-T, the blots were incubated with goat anti-mouse IgM (1:10,000 in TBS-T) as the secondary antibody for 3 hr at room temperature. After three washing steps*,* the ECL substrate was applied for 3 minutes. Finally, the bands were visualized and documented with the image analyzer software KODAK 1D 3.5.2 (Syngene, UK), and quantification of the band density was determined using by normalization to the respective β-actin band density [31].

### Immunohistochemistry

Rat pups (N = 5 for each group) were anesthetized *via* inhalation of ether on PD1 and 14, and were perfused transcardially with 0.9% saline, followed by 4% paraformaldehyde in 0.1 M phosphate buffer (pH = 7.4). The brains were fixed in 1% paraformaldehyde solution (pH = 7.4) for 2 days. After tissue processing and embedding in paraffin, 5 μm coronal serial sections were prepared using a rotary microtome (Leitz 1512, Germany). The boundary of hippocampus was defined in accordance with the atlas of Paxinos and Watson [32]. Then, 10 sections including the hippocampus from each animal were chosen by uniform random sampling and mounted on poly-L-lysine coated slides. The sections were deparaffinized with the xylene, rehydrated through descending concentrations of ethanol, and rinsed with 0.1 M phosphate buffer saline (PBS) for 10 min. The sections were treated with EDTA (pH = 8.4) in PBS at 37 °C for 15 min to retrieve antigen and immersed in a methanol/H_2_O_2_ solution (1:100) in the dark for 30 min to block endogenous peroxidases and rinsed 3 times, each for 5 min, in 0.05 M PBS plus 0.025% Trition X-100 at room temperature. The sections were then incubated in PBS containing 10% normal serum with 1% BSA for 2 hours at room temperature. In order to decrease the background staining, all the sections were treated with 10% normal goat serum as the host of the antibody in PBS for 30 min. The sections were then covered with the anti-PSA-NCAM mouse IgM monoclonal antibody (diluted 1:500 in PBS with 1% BSA) as the primary antibody in humidified chamber at 4°C overnight. Following the incubation period, the slides were washed extensively with PBS containing 0.025% Trition X-100 (5 min each, 3 times). After the wash, the sections were applied with HRP-conjugated secondary antibody (Goat anti-mouse IgM, Vector Laboratories, CA) diluted to 1:200 concentration in PBS with 1% BSA at room temperature for 2 hr. All the sections were washed extensively with PBS for 3 min and treated with diaminobenzidine (DAB) solution (0.03gr DAB in 100 ml PBS and 200 μl H_2_O_2_ per 100 ml PBS) at room temperature for 30 min in the dark. After washing the sections with running water, all sections were counterstained with a solution of Harris Hematoxylin for 1 min. Finally, the sections were dehydrated in increasing graded ethanol, cleared in xylene and mounted on glass slides. The immunostaining sections were evaluated and photographed by a light microscope (Olympus DP12, Japan). In order to detect the staining intensity, all of the reactions were observed by three examiners separately. On the basis of their staining intensity, the sections were graded as very weak, +; weak, ++; moderate, +++; strong, ++++ [33].

### Statistical analysis

The western blot data are reported as the mean ± SEM. The statistical analysis was performed using the one-way analysis of the variance (ANOVA). Following a significant P-value (*p*), the post hoc analysis (Tukey) was used to assess the specific group comparisons. Also the nonparametric analysis of Kruskal Wallis and Mann–Whitney tests were used to analyze the immunostaining scores. A significant difference was defined as *p* < 0.05.

## Results

In the present study, pregnant rats (both Kind^+^/Seiz^+^ and Kind^-^/Seiz^+^ groups) received three PTZ injections during GD14-19. In rats, development of hippocampus initiates from embryonic day 13 (E13), and E14-E20 is a critical period for neurogenesis and neural migration in the hippocampus [12,34,35]. Therefore, we choose GD14-19 for PTZ injections. Because hippocampal development normally continues until two weeks after birth in rat pups, we evaluated the effect of maternal epileptic seizures on PSA-NCAM expression in rat pups hippocampi on PDs 1 and 14.

### PTZ injection

During the first few PTZ-injections, the female rats exhibited some slight symptoms consisting of short-term shaking in the head and face (stage 1 of the Racine score). However, after repeated PTZ injections (an average of 13), they exhi bited tonic-colonic seizures associated with a lose of balance (stages 4 or 5 of the Racine score), (Table 1). The PTZ injection 10 days after the last kindling injection revealed that there was no significant difference in the intensity of symptoms from that observed after the last kindling injection in the same animals (*p* = 0.1).

**Table 1:** Number of PTZ injections required to reach each stage of the Racine score (Mean ± SD)

**Seizure Score**	**Stage 1**	**Stage 2**	**Stage 3**	**Stage 4**	**Stage 5**
No. of PTZ injections	2.2 ± 0.5	6.8 ± 0.7	9.8 ± 1.1	12.4 ± 0.8	13.6 ± 1.6

### Western blotting

**Figure 1:**
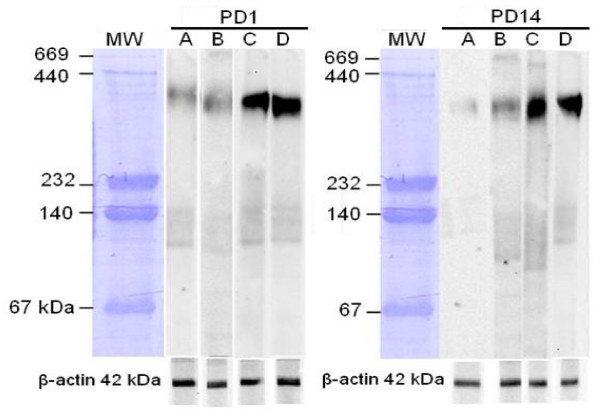
**Western blot analysis of PSA-NCAM expression in rat pups hippocampi on PD1 (left) and PD14 (right)**. A, Kind^+^/Seiz^+^ group; B, Kind^-^/Seiz^+^ group; C, Kind^+^/Seiz^-^ group; and D, the sham control group. The expression of PSA-NCAM in Kind^+^/Seiz^+^ and Kind^-^/Seiz^+^ groups has significantly decreased compared with the sham control group on both PD1 and PD14.

**Figure 2:**
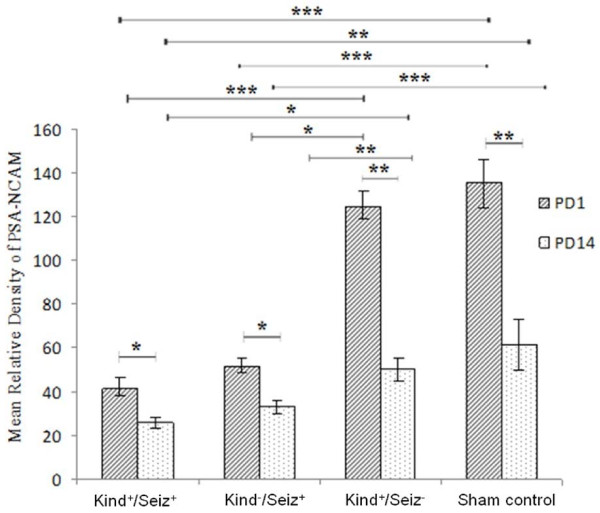
**The mean relative densities of PSA-NCAM expression on PD1 and PD14 (Mean ± S.D.)**. *: *p =* 0.01, **: *p =* 0.02, ***: *p =* 0.001.

After normalization against β–actin, the mean relative density of PSA-NCAM bands was calculated for all groups. As Figures 1 and 2 show, on PD1, the PSA-NCAM expressions of the offspring hippocampi for Kind^+^/Seiz^+^, Kind^-^/Seiz^+^, Kind^+^/Seiz^-^, and sham control groups were 41.07 ± 5.1, 51.34 ± 4.09, 124.61 ± 7.12, and 135.51 ± 10.52, respectively. By contrast, these values on PD14 were 25.89 ± 2.76, 32.86 ± 3.27, 50.08 ± 5.7, and 61.21 ± 11.47, respectively (Figure 2). This shows that PSA-NCAM expression significantly decreased in the hippocampi of the offspring born to mothers (both Kind^-^/Seiz^+^ and Kind^+^/Seiz^+^ groups) that experienced PTZ-induced seizures (stage 5 of the Racine score) during pregnancy, compared to the sham control group (*p* = 0.001). However, both on PD1 and PD14, PSA-NCAM expression in the hippocampus of rat pups born to Kind^+^/Seiz^-^ and sham control groups did not show any significant differences (*p* > 0.05), (Figure 2).

### Immunohistochemistry

**Figure 3:**
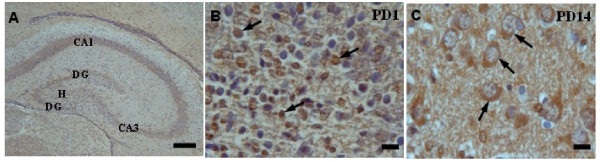
**Coronal sections of the rat pups hippocampus**: A) Different regions of sham control hippocampus; B) PSA-NCAM immunoreactivity in the hilus sub-region is restricted to the area around the nuclei in hippocampal neurons on PD1; C) a clear cytoplasmic layer around the nuclei of neurons in the hilus on PD14. Scale bars: A, 500 μm B; 50 μm; and C, 100 μm.

To evaluate the effect of epileptic seizures during pregnancy on the distribution of PSA-NCAM in the hippocampus, immunohistochemistry was performed. As shown in Figure 3, on PD1, the PSA-NCAM immunoreactivity is restricted to a thin layer around the nuclei of hippocampal neurons (Figure 3B). Whereas, on PD 14, the signal pattern changes to a clear cytoplasmic layer around the nuclei (Figure 3C).

**Figure 4:**
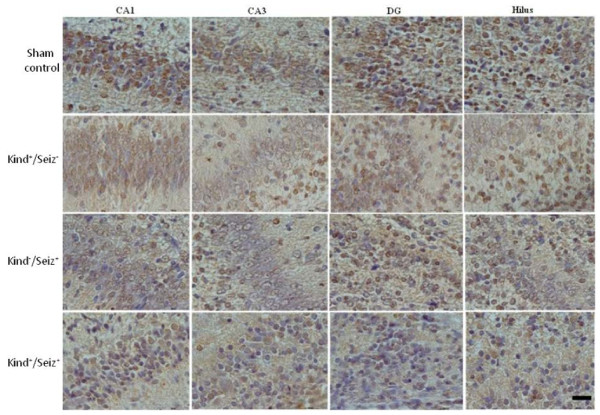
**Mean scores of PSA-NCAM immunoreactivity in different regions of rat pups hippocampus on PD1 (Mean ± SD)**. *: *p =* 0.01, **: *p =* 0.02, ***: *p =* 0.007.

**Figure 5:**
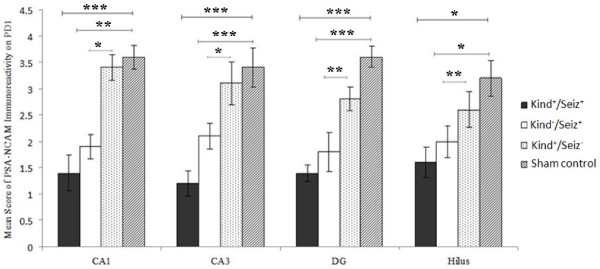
**Immunoreactivity of PSA-NCAM in different sub regions (CA1, CA3, DG, and hilus) of rat offspring hippocampus on PD1.** Scale Bar: 50 μm.

On PD1, PSA-NCAM positive cells are detected in the CA1, CA3, hilus, and dentate gyrus (DG) in the hippocampi of the sham control newborn rats (Figure 4). This pattern is similar in rat offspring hippocampi of sham control and Kind^+^/Seiz^-^ groups. By contrast, in the Kind^-^/Seiz^+^ and Kind^+^/Seiz^+^ groups, the PSA-NCAM immunoreactivity had more decreased in all parts of the hippocampus especially in the CA3 region (*p* = 0.007) in comparison to the sham controls (Figure 5).

**Figure 6:**
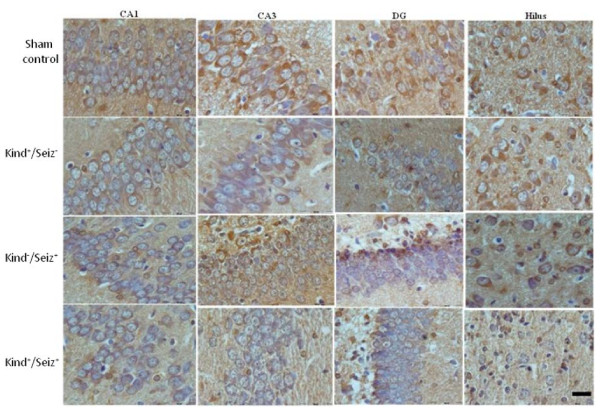
**Mean scores of PSA-NCAM immunoreactivity in different subregions of rat pups hippocampus on PD14 (Mean ± SD)**. *: *p =* 0.04, **: *p =* 0.02, ***: *p =* 0.009.

**Figure 7:**
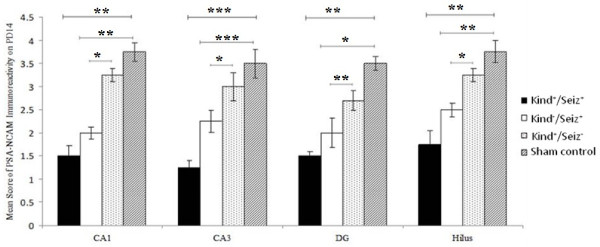
**Immunoreactivity of PSA-NCAM in different sub regions (CA1, CA3, DG, and hilus) of rat offspring hippocampus on PD14**. Scale Bar: 100 μm.

The PSA-NCAM immunoreactivity was highly evident in the pyramidal cell layer of the CA1 and CA3 regions, as well as the hilus and granular layer of DG rat hippocampus born to sham control group on PD14 (Figure 6). On PD14, the PSA-NCAM immunoreactivity was reduced in all regions, with an intensive decrease in the CA3 region, of the hippocampus in the rat pups of Kind^-^/Seiz^+^ and Kind^+^/Seiz^+^ groups (*p* = 0.009), (Figure 7). There were no significant differences observed in the PSA-NCAM immuno-staining between Kind^-^/Seiz^+^ and Kind^+^/Seiz^+^ groups (*p* = 0.1), (Figure 7). As shown in Figures 5 and 7, the lowest amount of PSA-NCAM immunoreactivity was observed in the hippocampi of the pups obtained from the Kind^+^/Seiz^+^ group.

## Discussion

The effect/s of seizures on PSA-NCAM expression is a controversial issue. There have been some reports showing that epileptic seizures increase the number of PSA-NCAM positive cells in the hippocampus of kindled rats as well as patients with temporal lobe epilepsy [27,36]. In contrast, other studies have reported that the number of PSA-NCAM positive cells severely decrease in pediatric and adult patients with temporal lobe epilepsy in comparison to the general population [37,38]. However, the authors were unable to define a reason for these discrepancies.

Although several studies have shown some effects of maternal seizures on the brain development [7,8,11,15], there has been no report concerning the effects of maternal seizures on PSA-NCAM expression in the offspring. In the present study, we assessed the effects of kindling either with or without seizure induction during pregnancy on PSA-NCAM expression in the rat offspring hippocampus on PD1 and 14. Our observations show that PTZ-induced seizures during pregnancy severely decrease the PSA-NCAM expression in the developing rat hippocampus born to both kindled and non-kindled mothers.

The results of our study illustrate that PTZ-induced seizures during pregnancy decrease PSA-NCAM immunoreactivity on PD1 in the CA3 region of Kind^+^/Seiz^+^ and Kind^-^/Seiz^+^ hippocampus by approximately 2.8 fold. The reductions in CA1, DG, and hilus subdivisions of hippocampus were 2.5, 2.5, and 2.3 fold compared to the sham control group, respectively. Similar results were observed on PD14. The hilus of dentate gyrus is more sensitive to seizure and ischemia during postnatal life in comparison to other regions of the hippocampus [39,40]. In adult rats, epileptic seizures mainly affected CA1 and CA3 neurons in comparison to other parts of hippocampus [28]. By contrast, the results of our study show that the CA3 region is more sensitive to maternal seizures during postnatal development. In a previous study, we have shown that maternal epileptic seizures significantly decreased the number of PSA-NCAM positive cells per unit area in the CA1, CA3, and DG subdivisions of the rat offspring hippocampus [41]. Thus, at least part of the reduction of PSA-NCAM expression detected in the western blotting experiments is likely a result of neuronal loss. However, the immunohistochemical results of our present study show a marked decrease of PSA-NCAM immunoreactivity in different subregions of the rat offspring hippocamus. Therefore, it could be concluded that the reduction of PSA-NCAM expression following PTZ-induced seizures during pregnancy is both a consequence of neuronal loss, at least in part, and a decrease of PSA-NCAM expression.

There is some evidence showing that seizures may cause aberrant neurogenesis *via* up-regulation of neuroblast markers such as Doublecortin (DCx), Collapsin response mediator protein-4 (CRMP-4), and PSA-NCAM, and are likely to contribute to network abnormalities in the formation of epileptic hippocampus [42,43]. The increased PSA-NCAM expression in the hippocampal neurons and also mossy fibers following seizures could also be due to an activity-dependent mobilization in pre-existing granule cells [43]. Finally, some reports suggest that ectopic neurogenesis after seizures appears to be a maladaptive and compensatory response [42,44,45]. However, the reduction of PSA-NCAM expression in the rat pups hippocampus by maternal seizures is probably mediated *via* different mechanisms such as hypoxia, acidosis, and increasing level of stress hormones both in the mother and fetus [7,46].

The reduction of PSA-NCAM expression and immunoreactivity in the hippocampus of rat pups that their mothers experienced PTZ-induced seizures during pregnancy on PD1 was similar to PD14. It can be concluded that maternal seizures probably induce decreased PSA-NCAM expression in the rat offspring hippocampus and this reduction persists until the two weeks after birth, which is a critical period in hippocampal development. Although PSA-NCAM expression tends to decrease in adults, a high level of PSA-NCAM expression occurs in the olfactory bulb, entorhinal cortex, hippocampus, hypothalamus, and subventricular zone in the adult brain [17,19].

It has been suggested that PSA-NCAM plays a key role in the survival of immature neurons and induction of neuroplasticity, both of which are essential for learning [47,48]. Because migrating and maturating hippocampal neurons are PSA-NCAM-positive, a decrease in PSA-NCAM expression could be a reflection of neurogenesis impairment in the hippocampus [48-50]. In fact, it has been reported that hippocampal activity and synaptic reorganization get impaired following reduction of PSA-NCAM expression [51]. PTZ-induced seizures during pregnancy cause intensive cognition impairment, learning/memory deficits, neurodegeneration, and also neurodevelopmental impairments in offspring during the postnatal period [9,36]. Indeed, it has been shown that maternal seizures could cause an imbalance between excitatory and inhibitory interneurons, as well as impaired neuronal migration in the rat offspring hippocampi, and probably induce abnormalities in the structure and function of the hippocampus [6,15].

Although the exact mechanism/s of the effect of maternal seizures on fetal development is not known [11], one of the effective factors is thought to be hypoxia. Hypoxia plays an important role in fetal brain defects [15,46,52]. A reduction of PSA-NCAM positive cells in rat pups' hippocampus after induction of hypoxia during the prenatal period has been reported previously [53]. It is likely that hypoxic-ischemic insults in the perinatal period reduced the number of PSA-NCAM positive cells in the subventricular zone of the rat offspring brain [54]. In fact, hypoxia triggers a cascade of biochemical changes including high Ca^2+^ concentrations (mechanism of nerve cell death in response to seizure), a decrease of detoxification enzymes activities, an increase of free radicals, and an increase of arachidonic acid metabolism and activation of inflammatory-like processes in the central nervous system that could lead to an irreversible neuronal damage [8,55].

Seizures during pregnancy also induce apoptosis and neurodegeneration by increasing caspase-3 activity and decreasing GABA_B1_ receptors in the hippocampus of rat pups [11]. Other effective factors, such as maternal trauma during tonic-clonic seizures and elevation of adrenal hormones level, should also be taken into consideration [7]. For example, it has been shown that increasing levels of glucocorticoids adversely affect PSA-NCAM expression in the rat hippocampus. In addition, under normal conditions, stress hormones suppress hippocampal PSA-NCAM expression [56,57].

## Conclusions

The results of our study show that epileptic seizures during pregnancy but not kindling: 1) adversely decrease PSA-NCAM expression in the rat pup’s hippocampus; 2) reduce the immunoreactivity of PSA-NCAM in different regions of the hippocampus; and 3) the reduction of PSA-NCAM expression persists until two weeks after birth, a critical period in rodent's hippocampus development. The observed maternal seizure-induced hippocampal PSA-NCAM expression disturbances might be considered as a probable factor for cognitive impairments, and learning/memory defects observed in children born to epileptic mothers. It is possible that there is a relationship between maternal seizures, PSA-NCAM expression, and structural and functional alterations in offspring hippocampi. However, further studies are needed to elucidate the molecular mechanism/s involved during prenatal and postnatal periods.

## Abbreviations

CA: Corna Amunis; DAB: Diaminobenzidine; DG: Dentate Gyrus; E: Embryonic Day; GABA: Gamma-Amino Butiric Acid; GD: Gestational Day; i.p: Intraperitoneally; PBS: Phosphate Buffer Saline; PD: Postnatal Day; PSA-NCAM: Polysialylated Neural Cell Adhesion Molecule; PTZ: Pentylenetetrazol.

## Competing interests

The authors declare that they have no competing interest

## Authors’ contributions

AR participated in the design of the study, and confirmation of the kindly and seizure scores. AE participated in the design of the study, helped to draft the manuscript and carried out the immunohistochemistry. AF conceived of the study, and participated in its design and coordination. MS carried out the western blotting. HR participated to manuscript revision and performed the statistical analysis. HH participated in the determination if hippocampus sub-regions and helped to draft the manuscript. All authors read and approved the final manuscript.
